# Lack of Liver X Receptors Leads to Cell Proliferation in a Model of Mouse Dorsal Prostate Epithelial Cell

**DOI:** 10.1371/journal.pone.0058876

**Published:** 2013-03-12

**Authors:** Julie Dufour, Aurélien Pommier, Georges Alves, Hugues De Boussac, Corinne Lours-Calet, David H. Volle, Jean-Marc A. Lobaccaro, Silvère Baron

**Affiliations:** 1 Clermont Université, Université Blaise Pascal, Génétique Reproduction et Développement (GReD), Clermont-Ferrand, France; 2 CNRS, UMR 6293, GReD, Aubiere, France; 3 INSERM, UMR 1103, GReD, Aubiere, France; 4 Centre de Recherche en Nutrition Humaine d’Auvergne, Clermont-Ferrand, France; Nihon University School of Medicine, Japan

## Abstract

Recent studies underline the implication of Liver X Receptors (LXRs) in several prostate diseases such as benign prostatic hyperplasia (BPH) and prostate cancer. In order to understand the molecular mechanisms involved, we derived epithelial cells from dorsal prostate (MPECs) of wild type (WT) or *Lxrαβ−/−* mice. In the WT MPECs, our results show that LXR activation reduces proliferation and correlates with the modification of the AKT-survival pathway. Moreover, LXRs regulate lipid homeostasis with the regulation of *Abca1*, *Abcg1* and *Idol*, and, in a lesser extent, *Srebp1*, *Fas* and *Acc*. Conversely cells derived from *Lxrαβ−/−* mice show a higher basal phosphorylation and consequently activation of the survival/proliferation transduction pathways AKT and MAPK. Altogether, our data point out that the cell model we developed allows deciphering the molecular mechanisms inducing the cell cycle arrest. Besides, we show that activated LXRs regulate AKT and MAPK transduction pathways and demonstrate that LXRs could be good pharmacological targets in prostate disease such as cancer.

## Introduction

Patient cohorts and human cancer cell lines give suitable biological samples to identify genetic defects correlated with disease but are still limited to investigate the molecular mechanisms involved. *In vivo* mouse models of prostate cancer represent a powerful tool to complement these human studies. A second advantage is that mice provide the possibility to derive long-term cell culture system genetically engineered. Hence, we have developed a useful cell culture system of prostatic epithelial cells (MPECs) from wild-type (WT) and transgenic mice with genetic ablation of both isoforms of the Liver X Receptor (*Lxr*). LXRα (NR1H3) and β (NR1H2) belong to the nuclear receptor superfamily and act as ligand-inducible transcription factors when heterodimerized with the Retinoid X Receptor (RXR, NR2B1 to B3). LXRs are involved in numerous physiological regulations such as cholesterol, fatty acids and glucose homeostasis, steroidogenesis and immunity [1], [2]. Various studies have highlighted the therapeutic potential of LXR agonists in the treatment of prostate diseases such as benign prostatic hyperplasia (BPH) [3], [4] and prostate cancer [5], [6]. Indeed, LXR agonists like T0901317 slow down proliferation of various prostate cancer cell lines and decrease growth of prostate cancer cells in xenografted nude mice [7]–[9]. This lower proliferation of human prostatic cancer cell line LNCaP is characterized by a reduced amount of cells in S phase correlated with an accumulation of SKP2, a kinase involved in the degradation of cell cycle inhibitors. This cell cycle regulation was proposed to be connected with cholesterol homeostasis through LXR-regulated expression of the cholesterol transporter ABCA1 that supports cholesterol efflux [10]. Additionally, LXR activation by the synthetic ligand T0901317 results in a decrease of cell survival. The lowering of cellular cholesterol induced by the cholesterol transporter ABCG1 accumulation leads to a reduction in lipid raft size inactivating the AKT pathway and consequently limiting cell survival ability [9]. These previous studies demonstrated a putative combined anti-proliferative and pro-apoptotic effect of LXR activation in prostate cancer cell lines, suggesting a protective role of these nuclear receptors in this cancer *via* their effects on the cell cycle and apoptosis balance [11].

Even if prostate is anatomically different between human and mouse since the rodent prostate is divided into distinct lobes whereas three zones are found in human [12], transcriptional analyses linked the mouse dorsolateral lobe to the human peripheral zone which develops cancer [13].

Here, we present an original prostate epithelial cell model derived from the dorsal lobe of WT or *Lxrαβ−/−* mice. This new cell models allowed deciphering the role of LXRs on cell cycle regulation.

## Materials and Methods

### Mouse Prostate Epithelial Cells (MPECs) Establishment

*Lxrα* and *Lxrβ* double knockout mice (*Lxrαβ−/−*) and their wild-type controls were maintained on a mixed strain background (C57BL/6∶129Sv) and housed in a temperature-controlled room with 12-h light, 12-h dark cycles. They were fed *ad libitum* with water and Global-diet 2016S (Harlan, Gannat, France). This study was carried out in strict accordance with the recommendations of French national standards and policies (D 63 104 19). Mice were killed by cervical dislocation and prostates harvested during necropsy in order to use in cell culture systems. The protocol was approved by local ethic committee - Permit Number: CE21-11 / CE75-12 (CEMEAA - Comité d’Ethique en Matière d’Expérimentation Animale Auvergne, https://www1.clermont.inra.fr/cemeaa/). The culture procedure was derived from that one used to develop the mouse vas deferens epithelial cells [14]. Briefly, mouse prostate epithelial cells (MPECs) were harvested from the dorsal prostate lobes from 20 to 30 day old mice, wild type (WT) or lacking both LXRs (*Lxrαβ−/−*), and transferred onto cell culture inserts (BD Falcon TM, Fontenay-sous-Bois, France) coated with a thin layer of extracellular matrix gel (Sigma Aldrich, L’isle d’Abeau, France). Cells were cultured in complete medium [Dulbecco’s modified Eagle’s medium (DMEM) /F12 (50∶50; Invitrogen, Oslo, Norway) supplemented with 0.5% fetal bovine serum (FBS) (Biowest, Nuaillé, France), cholera toxin (10 ng/ml), epidermal growth factor (5 ng/ml), penicillin and streptomycin (100 µg/ml), insulin (5 µg/ml), transferin (10 µg/ml), L-glutamine (2 mM), HEPES (20 mM), ethanolamine (0.6 µg/ml), cAMP (25 µg/ml), selenium (17.3 ng/ml) and hydrocortisone (10 nM)] at 37°C in a humidified air 5% CO_2_ incubator. The extracellular matrix gel was set by incubation at 37°C for 30 min. All the chemicals were from Sigma Aldrich unless indicated. Growth medium was changed every 2 days. Upon confluence, cells were re-plated in the same conditions at split ratio 1∶2 until they survived senescence, typically after six passages. After this point, cells were transferred onto 100 mm culture dishes every 3-4 days at a 1∶5 to 1∶10 split ratio.

### Genotyping

**Figure 1:**
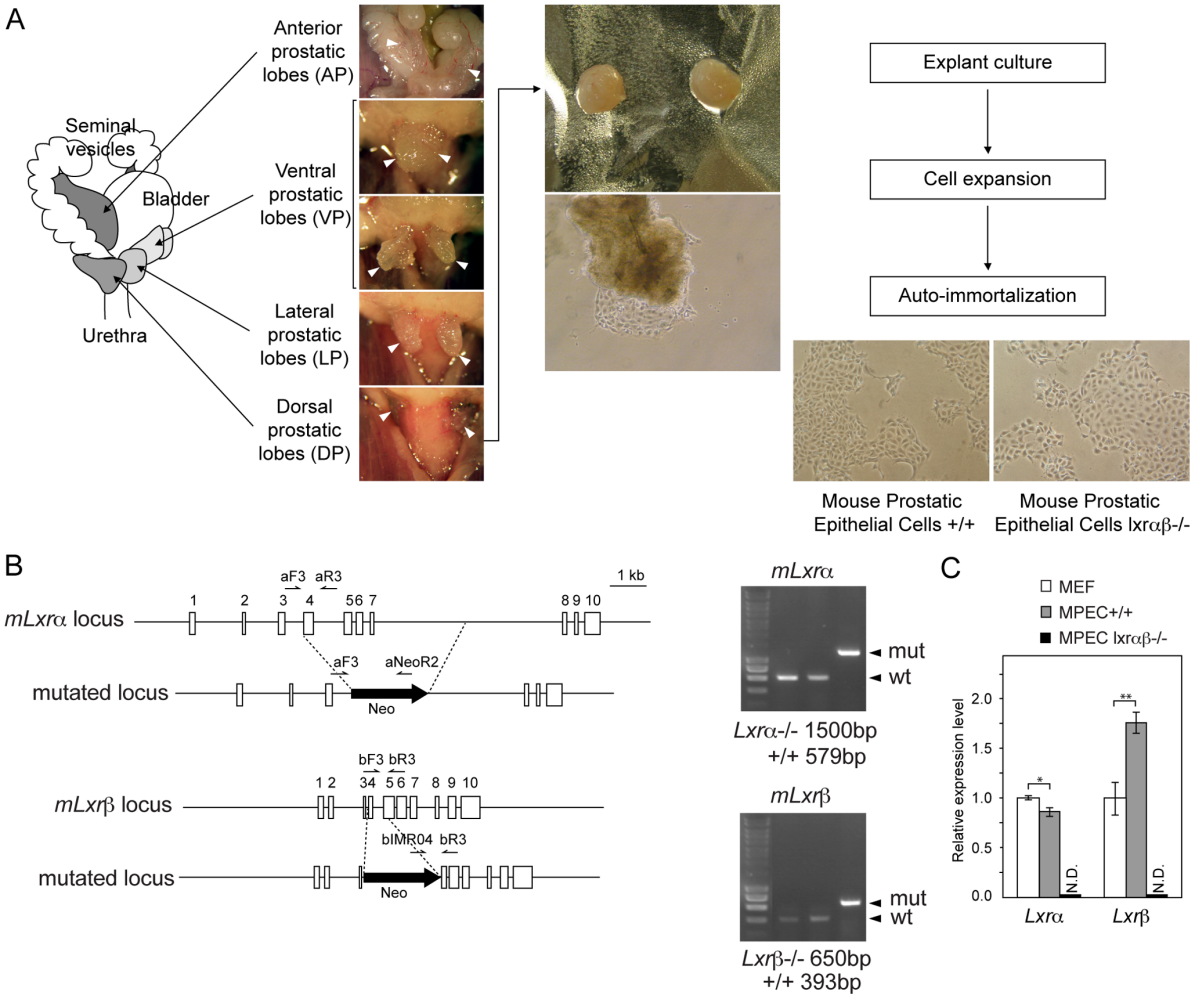
Establishment of WT and *Lxrαβ−/−* MPECs from dorsal prostate. (A) Schematic mouse prostate lobe representation (*left panel*). Dissected prostate sample pictures during necropsy (*middle panel*) and explant migration/primary epithelial cell culture (*right panel*). (B) Schematic representation of *Lxrα* and *Lxrβ* wild type and recombined genomic loci. Primer αF3, αR3, NeoR2 and βF3, βR3, IMR04 respectively used to genotype *Lxrα* and *Lxrβ* are depicted (previously described [24], D.J. Mangelsdorf personal communication). PCR analysis of WT and *Lxrαβ−/−* MPEC genomic DNA Lane 1: WT mouse embryonic fibroblasts (MEF); lane 2, WT MPECs; lane 3, *Lxrαβ−/−* MPECs. (C) qPCR analysis of *Lxrα* (*nr1h3*) and *Lxrβ* (*nr1h2*) accumulation in MPECs and MEFs, (N.D. not detectable). *p<0.05, **p<0.01, ***p<0.001 in Student’s *t* test. Error bars represent mean ± SEM. (qPCR analysis results from 4 independant experiments and was normalized using *36b4* gene).

*Lxrα* and *Lxrβ* loci with the neo cassette as well as the location of the primers are indicated on Fig. 1B. Primers used (kind gift from D.J. Mangeldorf, Dallas, TX) are for *Lxrα* forward primer αF3∶5′-ATGGAGAATGCCTAGCTAGG-3′ and two reverse primers, αR3∶5′-TCTCACTACGTAGCTCTTGG-3′ and NeoR2∶5′-AAGAACTCGTCAAGAAGGCGA-3′; for *Lxrβ* two forward primers βF3∶5′-CCTTTTCTCCCTGACACCG-3′ and IMRO4∶5′-AGGTGAGATGACAGGAGATC-3′ and a reverse primer βR3∶5′-GCATCCATCTGGCAGGTTC-3′. An example of PCR genotyping is shown in Fig. 1C. Presence of *Lxrα* and *Lxrβ* wild-type loci is characterized by a fragment of 579 and 393 bp, respectively; recombined *Lxrα* and *Lxrβ* loci show a fragment of 1500 and 650 bp, respectively.

### Reagents

22(R)-hydroxycholesterol and GW3965 were purchased from Sigma-Aldrich and T0901317 from Cayman Chemical (Montigny-le-Bretonneux, France). All ligands used in cell culture were diluted in DMSO. PD98059 (CAS 167869-21-8) was purchased from Millipore (Billerica, MA, USA) and LY-294002 (ST-420-0025) from Enzo Life Sciences (Villeurbanne, France).

### Cell Culture and Treatment

Mouse embryonic fibroblasts (MEFs) were cultured in DMEM supplemented with L-glutamine (2 mM) and penicillin streptomycin (100 µg/ml). MPECs were trypsinized and seeded at 3×10^5^ cells in 10 mm culture dishes. After 16h cells were starved for 24h in a minimal medium DMEM-F12 (50∶50, Invitrogen) supplemented with L-glutamine (2 mM) and penicillin streptomycin (100 µg/ml) and then with the same medium containing DMSO, T0901317, GW3965 or 22(R)-hydroxycholesterol for 48h. Cells were harvested and protein or RNA extraction, or flow cytometry analyses were performed.

### MTT Assay

MTT assay was performed according to manufacturer instructions (Sigma Aldrich). Briefly, cells were washed in PBS. MTT reagent (3-(4, 5-dimethylthiazol-2-yl)-2, 5-diphenyltetrazolium bromide, M 2128, Sigma Aldrich) was added and incubated at 37°C for 3 hrs. After MTT reagent removal, MTT solvent (10 % Triton 100X and 0.1 N HCL in anhydrous isopropanol) was added. Absorbance was determined in a Microwell plate reader (Model 680, Biorad, Marnes-La-Coquette, France).

### Flow Cytometry Analysis

Cells were recovered and washed in PBS. Pellets were resuspended in RNAse A (500 µg/ml), propidium iodide (50 µg/ml) solution and kept 1h at 4°C in the dark. Cell suspensions were analyzed using a Beckman Coulter fluorescence-activated cell sorter. At least 15,000 events were measured for each sample.

### Quantitative PCR

mRNA were extracted using the NucleoSpinRNAII kit (Macherey Nagel EURL, Hoerdt, France). cDNA was synthesized with 200U of Moloney murine leukemia virus-reverse transcriptase (Promega), 5 pmol of random primers (Promega), 40 U RNAsin (Promega) and 2.5 mM deoxynucleotide triphosphate. Quantitative PCR was performed on a Mastercycler ep Realplex (Eppendorf, LePecq, France) using MESA GREEN quantitative PCR masterMix Plus for SYBR (Eurogentec, Angers, France). Primer sequences are m*Lxrα* Fw: 5′-AGGAGTGTCGACTTCGCAAA-3′, m*Lxrα* Rev: 5′-CTCTTCTTGCCGCTTCAGTTT-3′; m*Lxrβ* Fw: 5′-AAGCAGGTGCCAGGGTTCT-3′, m*Lxrβ* Rev: 5′-TGCATTCTGTCTCGTGGTTGT-3′; m*Tenascin* Fw: 5′-GTCGTCTGGACACCAGGCCC-3′, m*Tenascin* Rev: 5′-CAGGGCCGGCATAGCCTTCG-3′; m*Snail* Fw: 5′-GTAACAAGGAGTACCTCAGC-3′, m*Snail* Rev: 5′-CTGGTATCTCTTCACATCCG-3′; m*Ck8* Fw: 5′-GGTGTCGGGGGCATCACAGC-3′, m*Ck8* Rev: 5′-CTGCCGGCGGAGGTTGTTGA; m*E-cadherin* Fw: 5′-ACGTCCATGTGTGTGACTGTG-3′, m*E-cadherin* Rev: 5′-AGGAGCAGCAGGATCAGAATC-3′; m*Abca1* Fw: 5′-GGAGCTGGGAAGTCAACAAC-3′, m*Abca1* 5′-ACATGCTCTCTTCCCGTCAG-3′; m*Abcg1* Fw: 5′-GCTGTGCGTTTTGTGCTGTT-3′; Rev: 5′-TGCAGCTCCAATCAGTAGTCCTAA-3′ ; m*Idol* Fw: 5′-AGCGGCCTCTACCGAGCCAT-3′, m*Idol* Rev: 5′-CGCCAAGTGGCCCTTCAGGT-3′; m*Srebp1* Fw: 5′-GCCTGTACAGCGTGGCTGGG-3′, m*Srebp1* Rev:5′-TCTCCGTCAGCTGCCCCTGG-3′; m*Acc* Fw: 5′-ACTTCCCGACCAAGGACTTTG-3′, m*Acc* Rev: 5′-ACAGTGGAGCTAGAATTGGAC-3′; m*Fas* Fw: 5′-CCCCAACCCTGAGATCCCA-3′, m*Fas* Rev: 5′-TTGATGCCCACGTTGCC-3′; m*36b4* Fw: 5′-GTCACTGTGCCAGCTCAGAA-3′, m*36b4* Rev: 5′-TCAATGGTGCCTCTGGAGAT-3′.

### Western Blot Analysis

Proteins were extracted in Hepes 20 mM, NaCl 0.42 M, MgCl_2_ 1.5 mM, EDTA 0.2 mM and NP40 1% supplemented with PMSF 1 mM (Sigma-Aldrich), Complete 1X (Roche Diagnostics, Meylan, France), NaF 0.1 mM, Na_3_VO_4_ 0.1mM and 0.5 mM DTT (Sigma-Aldrich). Lysates were centrifuged at 4°C for 15 min at 15000 g. Total proteins were subjected to denaturing SDS-PAGE and transferred to nitrocellulose Hybond-ECL membrane (GE Healthcare Life Sciences, Velizy-Villacoublay, France). Membranes were incubated overnight at 4°C with primary polyclonal antibodies raised against either human ABCA1 (NB400-105, Novus Biologicals, Littleton, USA), βActin (A2066, Sigma Aldrich), p42/44 (MS670, Sigma-Aldrich), AKT (#9272, Cell Signaling, Danvers, MA), PhosphoS473-AKT (#2118-1, Epitomics, Burlingame, USA), PhosphoT308-AKT (#2965, Cell Signaling), Gsk3β (#9315, Cell Signaling), PhosphoGSK3β (#9336, Cell Signaling), Pten (#9559, Cell Signaling), Bad (#9292, Cell Signaling), PhosphoBad (#4366S, Cell Signaling), Srebp1 (MS-1207-P, ThermoScientific, Brebières, France), Phospho-p42/44 (#4370, Cell Signaling), CK18 (H-80) (SC-28264, Santa-Cruz, Santa-Cruz, CA), PSCA (SC-28819, Santa-Cruz), p21 (SC-397, Santa-Cruz), p27 (SC-528, Santa-Cruz), CyclinE (SC-481, Santa-Cruz), cdk4 (SC-260, Santa-Cruz), CyclinD1 (SC-718, Santa-Cruz), Cdk2 (SC-163, Santa-Cruz), α-Tubulin (T6074, Sigma Aldrich). One hour incubation was done with peroxidase conjugated anti-rabbit IgG or anti-mouse (P.A.R.I.S, Compiègne, France) and detection performed using a Western Lightning System kit (PerkinElmer, Villebon sur Yvette, France).

### Cell Immunofluorescence

Cells were fixed in 4% paraformaldehyde and permeabilized in PBS Triton X-100 0.1%. Detections were performed using anti-CK8 (Covance, Princeton, NJ), anti-Ecadherin (Sigma Aldrich), anti-βtubulin (BD Transduction Laboratories, Le Pont de Claix, France), anti-PhosphoS473-AKT (Epitomics) and revealed with Alexa 488-conjugated anti-rabbit and Alexa 555-conjugated anti-mouse immunoglobulins (Invitrogen). Slides were mounted with PBS/glycerol and were visualized with a Carl Zeiss Axiocam digital camera on a Zeiss Axioplan 2 microscope.

### Lipid Analysis

After permeabilisation with triton-X100 10%, lipid staining was performed using Oil-Red-O (Sigma-Aldrich) as already described [15].

### Statistical Analysis

Values are expressed as means ± SEM. Statistical comparisons were performed using a two-tailed Student’s *t* test. A p< 0.05 was considered statistically significant.

## Results

### Establishment of MPECs from Mouse Dorsal Prostate Lobe Explant

To generate MPEC culture, dorsal prostate lobes were dissected from wild-type (WT) and *Lxrαβ−/−* mice (Fig. 1A). Prostate samples were minced and seeded onto ECM-coated plates in epithelial specific defined-medium (procedure details were recapitulated in *Methods* section). Cells migrated from the explant and culture was maintained until auto-immortalization of epithelial cells. Upon immortalization, cell genotypes were checked by PCR (Fig. 1B), and *Lxrα* and *Lxrβ* transcript accumulations were determined and compared to WT MEFs (Fig. 1C). It appears that *Lxrα* slightly shows similar levels between MPECs and MEFs, while *Lxrβ* is more accumulated in MPECs than in MEFs. As expected, both messengers were undetectable in *Lxr−/−* MPECs.

**Figure 2:**
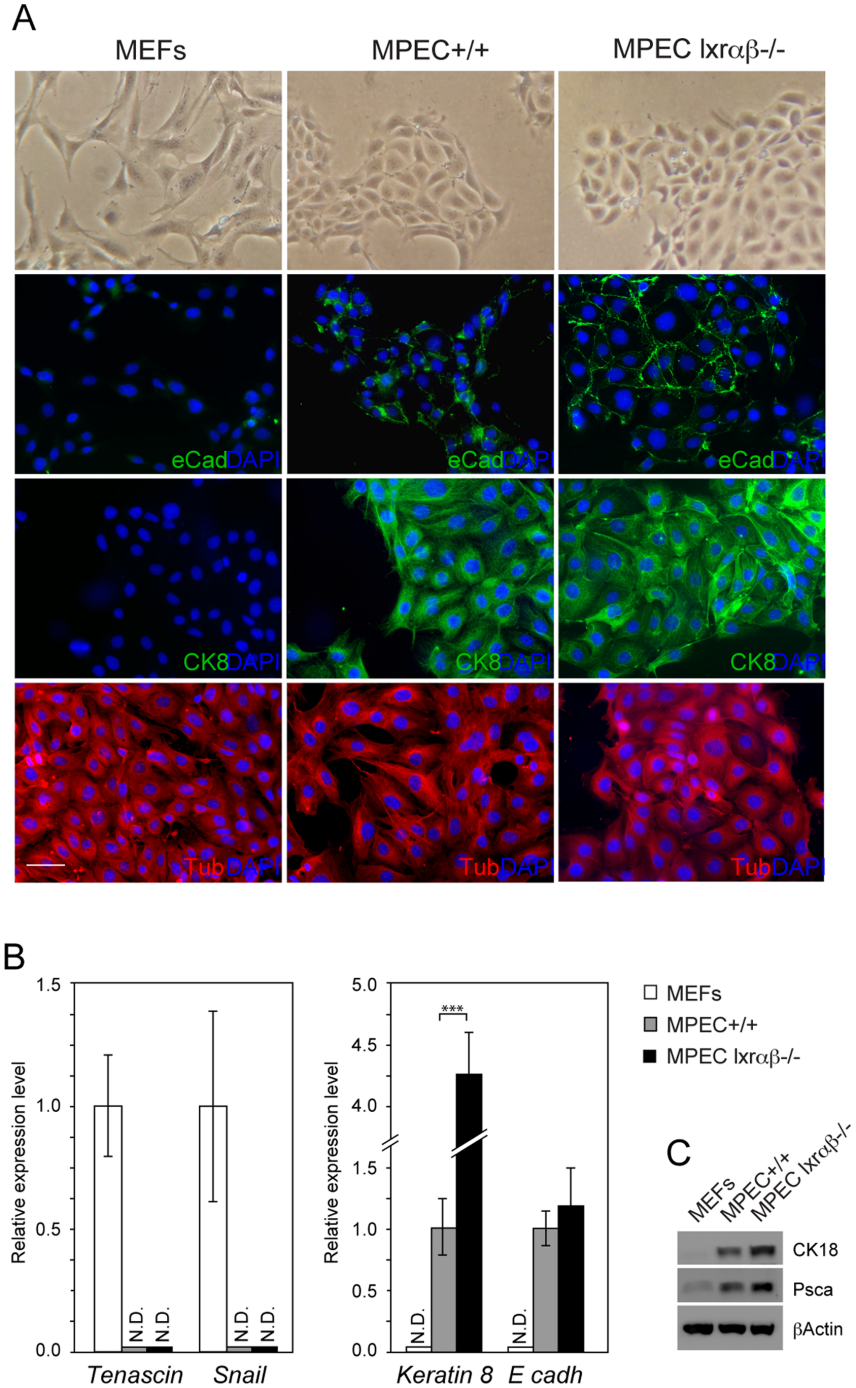
Analysis of stromal and epithelial markers in WT or *Lxrαβ−/−* MPECs. (A) WT and *Lxrαβ−/−* MPECs and WT MEFs were immunostained using anti-E-cadherin (E-Cad), anti-Cytokeratin8 (CK8) and anti-αTubulin (Tub) antibodies. Scale bar 100 µm. (B) mRNA relative levels of *Tenascin, Snail, Keratin 8* and *E-cadh* were measured in WT MEFs, WT (+/+) and *Lxrαβ−/−* MPECs by qPCR. *p<0.05, **p<0.01, ***p<0.001 in Student’s *t* test. Error bars represent mean ± SEM. (qPCR analysis results from 3 independant experiments and was normalized using *36b4* gene) (C) Western blot analysis was performed on WT MEFs, WT (+/+) and *Lxrαβ−/−* MPECs using CK8, Psca antibodies. βActin was used as a loading control.

Once cultured, MPECs formed islands and displayed typical cobblestone features of epithelial cells compared to spindle shape of MEFs (Fig. 2A). In order to check for contamination of the culture by stromal fibroblasts, stromal and epithelial differentiation markers were investigated. Absence of both *Tenascin* and *Snail* expressions avoid potential contamination by mesenchymal cells (Fig. 2B). Immunofluorescence and qPCR detection of specific markers *E-cadherin* and *Cytokeratin 8* confirmed the epithelial origin of the culture (Fig. 2A,B), and as expected these markers were absent in MEFs. The epithelial characteristics of the MPECs were strengthened by accumulation of CK18 and PSCA proteins (Fig. 2C). We concluded that MPECs could be considered as a good tool to study the physiological role of LXRs in epithelial cells from dorsal prostate.

### LXRs Control Cell Proliferation in MPECs

**Figure 3:**
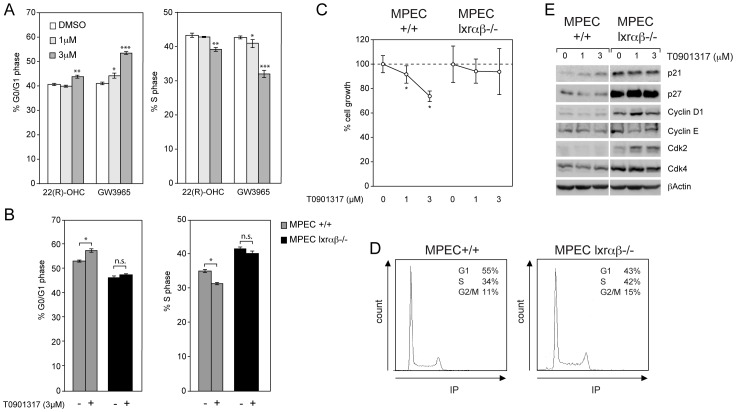
LXRs are involved in the control of MPEC proliferation. (A) Percentage of WT MPECs in G0/G1 phase and S phase after DMSO (vehicle), 1 and 3 µM 22(R)-hydroxycholesterol (22(R)-OHC) or GW3965 treatments was quantified by flow cytometry. N = 3 (B) Percentage of WT (+/+) and *Lxrαβ−/−* MPECs in G0/G1 and S phase after DMSO (vehicle) or T0901317 (3 µM) treatments was quantified by flow cytometry. (C) Percentage of WT (+/+) and *Lxrαβ−/−* MPEC cell growth determined by MTT assay after DMSO (vehicle) or T0901317 1 and 3 µM treatments. *p<0.05, **p<0.01, ***p<0.001 in Student’s *t* test. Error bars represent mean ± SEM. N = 3 (D) Flow cytometry profile of WT (+/+) and *Lxrαβ−/−* MPECs in basal condition. (E) Protein lysates from WT (+/+) and *Lxrαβ−/−* MPECs incubated with DMSO or 1, 3 µM T0901317 for 48h were analyzed by western blot using antibodies raised against p21, p27, Cyclin D1, Cyclin E, Cdk2 and Cdk4. βActin was used as loading control.

LXR agonists have been previously described to exert anti-proliferative activities [16]. Thus, we investigated the effect of *Lxr*-ablation in MPECs. Treatments with various concentrations of 22R-hydroxycholesterol, a natural LXR-ligand, or GW3965, a synthetic LXR-ligand, on WT MPECs result in a dose-dependent accumulation of cells in G0/G1 phases and conversely a decrease of the percentage of cells in S phase (Fig. 3A). These observations show that the effect of the ligands on cell cycle is also visible in non-cancerous cells and is rather an intrinsic property of prostate epithelial cells. The effect of T0901317 (synthetic LXR ligand) was analyzed on *Lxrαβ−/−* MPECs and, as expected, had no effect on cell cycle (Fig. 3B) and proliferation (Fig. 3C). Interestingly, *Lxrαβ−/−* MPECs exhibit a higher basal proliferation (Fig. 3B and D). LXRs are thus regulators of cell cycle, which correlates with Cyclin D1 and Cdk2 protein accumulation (Fig. 3E). While GW3965 or T0901317 treatment decreases proliferation in WT MPECs, these ligands have no effect on *Lxrαβ−/−* MPECs (Fig. 3B). It could thus be concluded that GW3965 or T0901317 cell cycle inhibition is strictly LXR-dependent in MPECs, without potential interferences with other nuclear receptors. Interestingly, although T0901317 induction increased p21 cell-cycle inhibitor accumulation in WT MPECs that is consistent with proliferation decrease, *Lxrαβ−/−* MPECs reached a high and constitutive level of p21 that contrasts with the high proliferative rate (Fig. 3E). Paradoxically, p21 targets Cdk2/4, CyclinD1 and CyclinE were increased in *Lxrαβ−/−* MPECs indicating that driving forces leading to proliferation overcome p21 regulation. Concomitantly to p21 deregulation in *Lxrαβ−/−* MPECs, p27, initially identified as a downstream target of LXR activation in LNCaP cells [7], exhibit a strong accumulation as well. As observed in prostate cancer PTEN-null mice model, we postulated that p21 and p27 accumulation level in *Lxrαβ−/−* MPECs could result from a defensive mechanism to counterbalance the proliferation increase due to the combined lack of *Lxrα* and *Lxrβ* [17].

### LXRs and Lipid Homeostasis in MPECs

**Figure 4:**
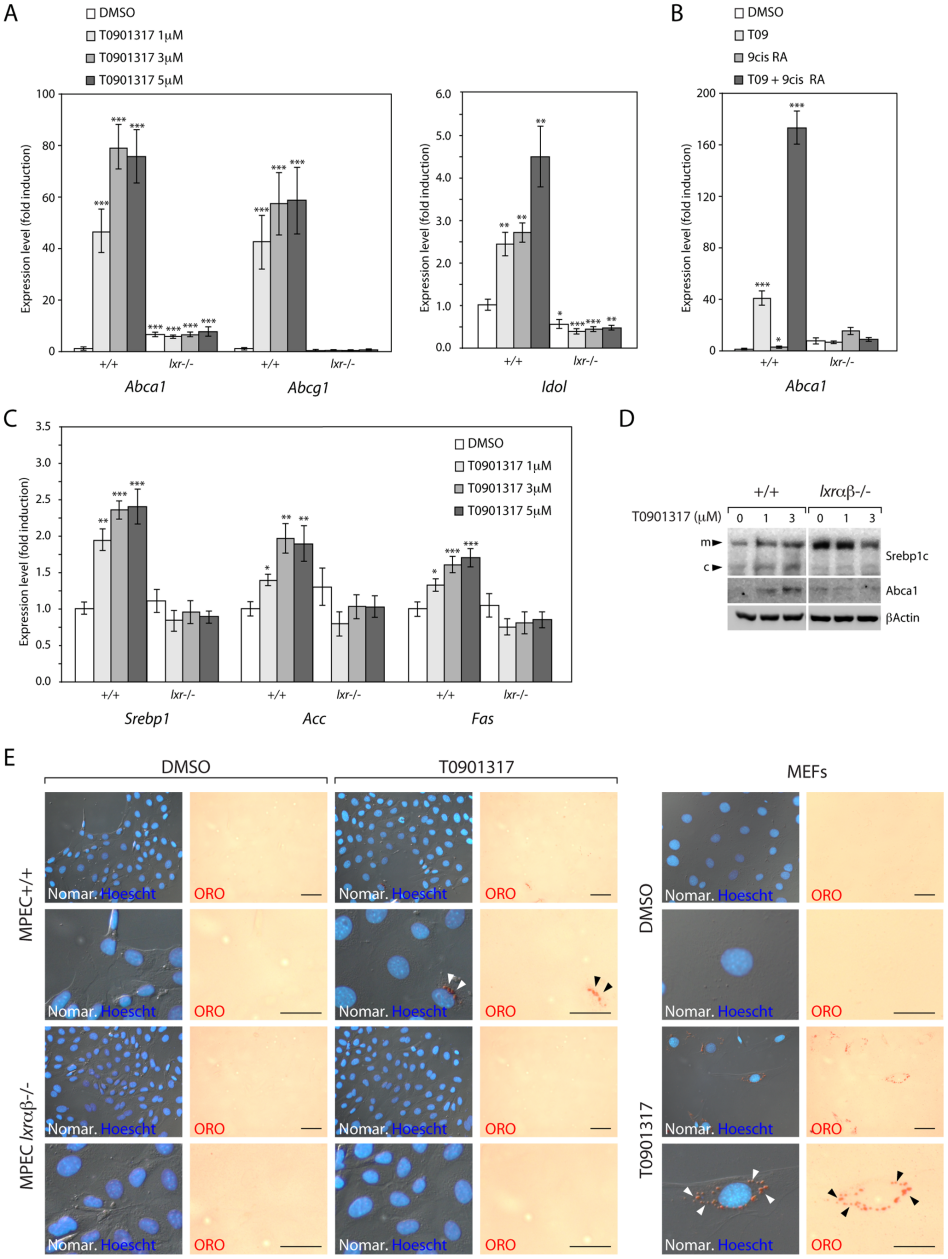
LXRs control expression of genes involved in cholesterol homeostasis and fatty acid synthesis in MPECs. (A) qPCR analysis of *Abca1*, *Abcg1* and *Idol* levels in WT (+/+) and *Lxrαβ−/−* (lxr*−*/*−*) MPECs after DMSO (vehicle) or T0901317 stimulation (B) Effect of 9-*cis* retinoic acid and/or T0901317 stimulation on *Abca1* accumulation levels in WT (+/+) and *Lxrαβ−/−* (lxr*−/−*) MPECs (C) qPCR analysis of *Srebp1*, *Acc*, and *Fas* levels in WT (+/+) and *Lxrαβ−/−* (lxr*−/−*) MPECs after DMSO (vehicle) or T0901317 stimulation. (qPCR analysis results from 4 independant experiments and was normalized using *36b4* gene). *p<0.05, **p<0.01, ***p<0.001 in Student’s *t* test. Error bars represent mean ± SEM. (D) Western blot analysis was performed on WT (+/+) or *Lxr*αβ*−/−* MPECs using Srebp1c, Abca1 and β-Actin antibodies. (E) Oil-Red O staining (ORO) and Normarski/Dapi of WT (+/+) and *Lxrαβ−/−* MPECs, or WT MEFs, treated for 48h with DMSO (vehicle) or T0901317 (1 µM). Head arrows indicate lipid droplets. Scale bars 100 µm.

As LXRs are transcription factors involved in cholesterol and fatty acids metabolism regulation, we have analyzed the effects of LXRs on these metabolic pathways. WT and *Lxrαβ−/−* MPECs were treated with increasing amounts of T0901317 and expression of well-known LXR target genes was analyzed by qPCR. As expected, WT MPECs displayed increased accumulation of *Abca1* and *Abcg1*, encoding cholesterol transporters, after LXR activation (Fig. 4A, left). A higher basal level of *Abca1* was observed in *Lxrαβ−/−* MPECs, as already described [18]. Conversely, no increased accumulation of *Abca1* and *Abcg1* was seen in *Lxrαβ−/−* MPECs. *Idol* whose product controls the degradation of LDL-receptor (LDLR) is induced by T0901317 (Fig. 4A, right), indicating that the LXR target genes involved in cholesterol homeostasis are inducible in MPECs. Co-stimulation of LXR/RXR heterodimer by T0901317 and 9-*cis* retinoic acid reproduces the canonical synergistic transcriptional activation of *Abca1* expression (Fig. 4B), thus demonstrating that LXR-signaling pathway is fully functional in immortalized MPECs. Interestingly, LXR-target genes involved in fatty acid synthesis such as *Srebp1c*, *Acc* and *Fas* showed a modest but significant increase in their expression when LXRs are activated in WT MPECs (fold range induction ×1.7 to x2.4) (Fig. 4C). These findings were correlated with western blot analyses in which WT MPECs displayed increased protein accumulation of ABCA1 and the non-cleaved form of SREBP1c in response to T0901317, conversely to what was observed in *Lxrαβ−/−* MPECs (Fig. 4D). Altogether, expression profiles of genes involved in lipogenesis postulate for a tenuous storage of triglycerides in MPECs in response to LXR activation. Oil red-O staining confirm that point since a few number of cells exhibits lipid droplets filling (Fig. 4E) in WT MPECs compared to MEFs treated with T0901317 (Fig. 4F). These results show that in WT MPECs, LXRs can be efficiently activated and subsequently reproduces canonical LXR mediated lipid metabolism regulation.

### LXRs are Connected to AKT and MAPK Transduction Pathways

**Figure 5:**
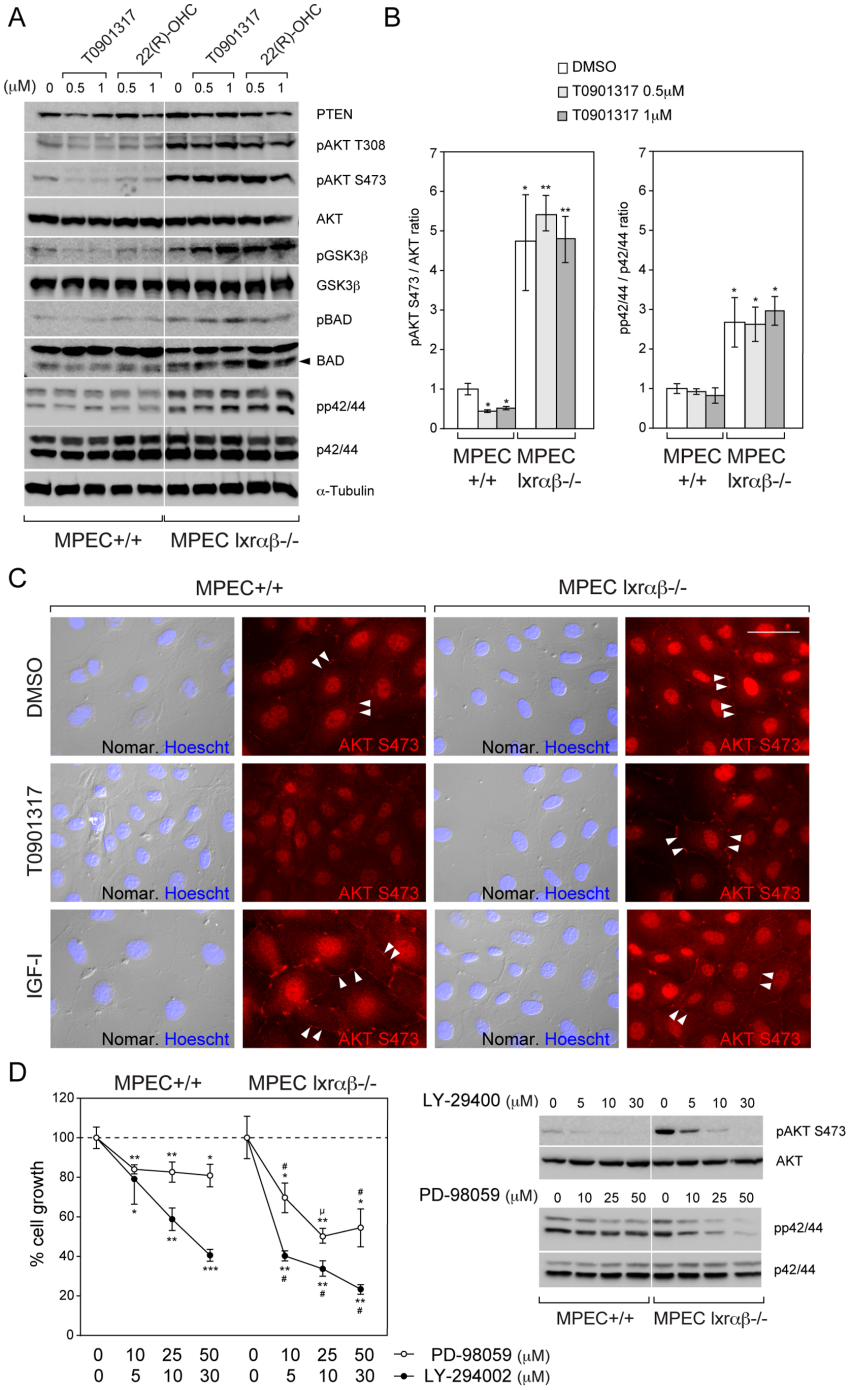
LXRs are necessary to warrant proper transduction pathway activities. (A) Western blot analysis was performed on WT (+/+) or *Lxr*αβ*−/−* MPECs, treated with DMSO (vehicle), T0901317 or 22(R)-hydroxycholesterol (22(R)-OHC), using antibodies against PTEN, pAKT S473, pAKT T308, AKT, pGSK3β, GSK3β, pBAD, BAD, pp42/44, p42/44 and α-Tubulin as a loading control. (B) Quantification of pAKT S473 / AKT and pp42/44 / p42/44 ratios of WT (+/+) or *Lxr*αβ*−/−* MPECs, treated with DMSO (vehicle) or T0901317 (N = 3). (C) WT (+/+) and *Lxrαβ−/−* MPECs were treated with DMSO (vehicle), T0901317 (1 µM) or IGF-1 (50 ng/ml) as a control and immunofluorescence was observed by microscopy using pAKT S473 antibody (nuclear staining is nonspecific). Scale bars 100 µm. (D) MTT assays were performed on WT (+/+) or *Lxr*αβ*−/−* MPECs, treated with DMSO (vehicle), LY-294002 (5, 10, 30 µM) or PD-98059 (10, 25, 50 µM) inhibitors. *p<0.05, **p<0.01, ***p<0.001 in Student’s *t* test *vs.* DMSO treatment, # p<0.01, µ p<0.001 in Student’s *t* test *vs.* WT (+/+) MPECs. Error bars represent mean ± SEM. N = 3. Treatment efficiency was checked by western blot using antibodies against, pAKT S473, AKT, pp42/44 and p42/44.

Previous studies showed that LXR activation could impact transduction pathways such as PI3K/AKT signaling in human prostatic cancer cell lines [9]. To examine whether such regulation was still present in MPECs, we monitored various protein accumulations and/or phosphorylations involved in this transduction pathway. Both AKT phosphorylation of Serine 473 (pAKT S473) and Threonin 308 (pAKT T308) are sensitive to T0901317 or 22(R)-hydroxycholesterol treatment, as described in LNCaP cells (Fig. 5A,B). As expected this sensitivity was not seen in *Lxr-*deficient cells (Fig. 5A,B). However it has been puzzling to observe that AKT phosphorylation level was basally found substantially higher (Fig. 5A,B) and insensitive to IGF-I (Fig. 5C and data not shown) in *Lxrαβ−/−* MPECs. In order to investigate whether AKT phosphorylation modifications induced by LXR stimulation or ablation was relevant, we monitored phosphorylation levels of GSK3β and Bad, two known downstream targets of AKT. As expected, both proteins exhibited a decreased phosphorylation in WT MPECs treated with T0901317 or 22(R)-hydroxycholesterol and a strong increased of basal phosphorylation in *Lxrαβ−/−* MPECs (Fig. 5A). These results were paralleled by immunofluorescence detection of phosphorylated AKT. Indeed a similar membrane staining to those observed in LNCaP cells [9] could be seen. This signal was lost in WT MPECs incubated with T0901317, but remained persistent in *Lxrαβ−/−* MPECs whatever the conditions. Abnormal transduction signaling in *Lxrαβ−/−* MPECs is not restricted to AKT pathway as phosphorylation forms of MAPK (pp42/pp44) were also basally found highly accumulated in these cells (Fig. 5A,B). These observations on PI3K/AKT and MAPK transduction pathway deregulations suggest that they could sustain proliferation activity and act as mediators of the LXR effects. Indeed, incubation of WT and *Lxrαβ−/−* MPECs in a medium supplemented with increasing amounts of pharmacological inhibitors of PI3-Kinase (LY-294002) or MEK (PD-98059) leads to a strong inhibition of cell growth. Consistent with the deregulated activity of AKT and MAPK in *Lxrαβ−/−* compared to WT MPECs, *Lxrαβ−/−* cells exhibit a higher sensitivity to both LY-294002 and PD-98059 treatments in MTT assays (Fig. 5D). Taken together, these results provide evidences that LXRs are important regulators of PI3K/AKT and MAPK transduction pathways, two central crossroads that mediate cell cycle control in MPECs.

## Discussion

This study characterizes MPECs derived from WT and *Lxrαβ−/−* mice. Our results indicate that LXRs are necessary to restrain cell cycle in non-cancerous prostate cells. Moreover, various LXR agonists, *e.g.* T0901317, GW3965 and 22(R)-hydroxycholesterol, clearly exert an anti-proliferative effect through LXRs, excluding the involvement of other nuclear receptors in the process we identified in MPECs. Molecular links between LXR target genes and proliferation process remains however to be elucidated. In the present study, we show that LXRs have a crucial role in the regulation of genes involved in cholesterol homeostasis and in a lesser extent in fatty acid synthesis in MPECs. Finally, we show that LXRs modulate AKT and MAPK phosphorylation accumulation, making them potential mediators of LXRs in the control of cell cycle.

LXRs were shown to modulate cell proliferation when they are activated by synthetic or natural ligands in prostate tumor cell lines [7]. Nevertheless, several studies questioned the specificity of the ligands used. Thus, some of these ligands have been proposed to interfere with distinct nuclear receptors [19]. In *Lxrαβ−/−* MPECs, none of the tested LXR ligands (T0901317, GW3965) displayed modulation of proliferation, demonstrating that LXRs are involved in the control of cell cycle in this context.

The results raise the question of the link between LXRs and the cell cycle effectors. Fukuchi *et al.* suggested ABCA1 to be a key-regulator of the cell cycle in response to LXR-activation in LNCaP cells [10] and suspected that cholesterol efflux is of major importance in this context. Consistent with these previous observations, LXR-target genes involved in cholesterol homeostasis (*e.g. Abca1*, *Abcg1* and *Idol*) were found up-regulated in WT MPECs in response to LXR ligands. These facts indicate that MPECs are responsive to cholesterol network homeostasis such as efflux/uptake activities. To date, various studies identified fatty acid metabolism as a crossing point in prostate tumor cell lines that is tightly associated to cell growth and proliferation [20]–[22]. Indeed, inhibition of acetyl CoA carboxylase (ACC) by Soraphen A efficiently blocks the proliferation of LNCaP cells, showing that tumor cell growth is dependent of fatty acid synthesis [23]. In MPECs, *Srebp1*, *Fas* and *Acc* display a significant but modest induction. Accordingly, the weak neutral lipid accumulation of MPECs (Fig. 5B) correlated the fact that induction of the fatty acid synthesis gene program is poorly sensitive to LXR regulation in these cells. Futhermore, the present work suggests that the capacity of LXRs to modulate cell cycle in MPECs is mostly dependent of their regulation of cholesterol homeostasis.

Unexpectedly, we observed a higher basal phosphorylation of AKT in MPECs lacking LXRs. It could be postulated that AKT signaling perturbation is a consequence of cholesterol metabolism alteration in *Lxrαβ−/−* MPECs. This hypothesis is consistent with the previous study demonstrating that LXRs control AKT phosphorylation levels in a raft-dependent manner in LNCaP cells [9].

Altogether, we show that LXRs *per se* modulate cell cycle in non-cancerous epithelial cell model. *Lxrαβ−/−* MPECs provide thus a powerful tool to investigate intrinsic connections between oxysterols, cholesterol metabolism and cell proliferation.
